# Effect of Second and Third Molar Eruption Stages on First Molar and Maxillary Arch Distalization With Modified Palatal Anchorage Plate and Beneslider: A 3D Finite Element Analysis

**DOI:** 10.7759/cureus.61403

**Published:** 2024-05-31

**Authors:** Siva Prasad Sripathi, Revathi Peddu, Ashok Kumar Talapaneni, Kalyani Mallavarapu, Aruna Dokku, Devikanth Lanka, Saravanan Pichai, Souren Bellam, Shaik Asma Bhanu, Lakshmi Kalyani

**Affiliations:** 1 Department of Orthodontics, Sibar Institute of Dental Sciences, Guntur, IND; 2 Department of Medicine, NRI Medical College and Hospital, Guntur, IND

**Keywords:** en-masse maxillary arch distalization, finite element analysis, modified palatal anchorage plate, distalization, beneslider

## Abstract

Aim: To analyze the effects of the maxillary second molar and third molar eruption stages on the distalization of first molars with a modified palatal anchorage plate (MPAP) and Beneslider using three-dimensional (3D) finite element analysis.

Materials and method: Six finite element models (FEMs) of individual maxillary molar distalization and six FEM models of en-masse maxillary arch distalization (EMAD) at different stages of the maxillary molar eruption were created from cone-beam computed tomography (CBCT) images of the maxillary complex, and 3D displacements of the maxillary first and second molars were evaluated with MPAP and Beneslider.

Results: On individual molar distalization, Beneslider showed first molar distal translation during the second and third molar follicular stages, while MPAP showed distal tipping of the first molar. With EMAD, either of the appliances showed distal tipping of the first molars. There was palatal rolling and extrusion of the first molars. The second molar showed buccal drifting with intrusion, and the incisors showed palatal displacement along with extrusion.

Conclusions: Second and third molar eruption stages had no adverse influence on first molar and en-masse maxillary arch distalization. Beneslider showed distal translation of the first molar, while distal tipping was seen with MPAP.

## Introduction

A paradigm shift towards a non-extraction treatment modality led the clinician to choose maxillary molar distalization to gain space for resolving moderate anterior crowding and to correct the Class II molar relation. Maxillary molar distalization can be achieved using either intraoral or extraoral appliances. However, these appliances have the unwanted side effect of distal tipping of molars and anterior anchorage loss in the form of incisal proclination.

Noncompliant distalization of maxillary molars as well as en-masse maxillary arch distalization (EMAD) is performed using mini-implant-aided mechanics to compensate for the problems and side effects of intraoral and extraoral distalization appliances [1-7]. Another biomechanical advantage of mini-implant-aided mechanics is that the point of force application passes through the Center of Resistance (CR) of the maxillary molar, resulting in nearly translational distal movement of the maxillary molar [8]. Temporary skeletal anchorage devices (TSADs) are applied to the buccal plate, palatal, and zygomatic bones to achieve individual molar and entire maxillary distalization [4,9-11]. However, mini-implants on the buccal cortical plate with minimal inter-radicular space limit the amount of tooth movement [12]. A finite element (FE) study showed that distalization with a palatal plate provided more bodily movement compared to buccally placed mini-implants [13]. Kook et al. reported an appliance called modified palatal anchorage plate (MPAP) to be effective in molar distalization with minimal distal tipping of molars (3.4 degrees) and its versatility in the application of force close to the vault of the palate achieved distal translation and intrusion of the molar and the authors recommended the use of MPAP in total arch distalization [12]. Wilmes et al. introduced a molar distalization appliance and reported almost translational movement of a molar with only 1.9 degrees of tipping, attributing this effect to the force vector close to the center of resistance of the molar [14,15].

Several studies have suggested that second and third molars are potential sources of resistance to the distalization of first molars. Gianelly et al. recommended the extraction of maxillary third molars before distalization of posterior teeth [16]. Higher rates of movement and greater distal crown tipping were reported when the second molars were at the apical third of the first molars than when the second molars had erupted in several studies [17-19]. In contrast, certain authors reported the minimal effect of the eruption status of second and third molars on first molar distalization [20-22]. In their finite element study, Kang et al. demonstrated greater distal root movement than a crown movement with MPAP, with an erupting second molar [23]. There is ambiguity regarding the influence of the eruption stage of second and third molars, and no studies have compared the efficacy of palatally anchored MPAP and Beneslider. 

Hence, this study aims to evaluate and compare the efficacy of MPAP and Beneslider on first molar distalization and en-masse distalization under the influence of maxillary second and third molar eruption stages using three-dimensional finite element analysis.

## Materials and methods

Cone-beam computed tomography (CBCT) images of the maxillary complex were used to generate a finite element (FE) model. The CBCT pictures were utilized to create a 3D model using STL Software (MIMICS 8.11 software, Materialise NV, Belgium). The 3D data were updated with HyperMesh V11 Software (Altair® HyperMesh®; Altair Engineering, Troy, MI) to create a tetrahedral FE mesh, with the maxilla, teeth, and alveolar bone meshing into 1-mm tetrahedrons and the rest of the skull, except the maxilla, meshing into 5-mm tetrahedrons. Teeth, alveolar bone, and the periodontal ligament (PDL) were all thought to be homogeneous and isotropic. Frictionless encounters between the teeth would be expected. The PDL's thickness is estimated to be 0.2 millimeters. Miniscrews were inserted into the anterior and posterior paramedian palatal bones. Table 1 shows the mechanical properties of the bone, teeth, miniscrews, stainless steel (SS) wire, and PDL in the model [24,25].

**Table 1:** Young’s modulus and Poisson’s ratio of various materials. PDL: periodontal ligament, SS: stainless steel.

Material	Young’s modulus (MPa)	Poisson’s ratio
Cortical bone	1.37 × 10^4^	0.30
Cancellous bone	7.90 × 10^3^	0.30
Palatal plate	1.05 × 10^5^	0.33
Miniscrew	1.05 × 10^5^	0.33
Tooth	2.07 × 10^4^	0.30
SS wire	2.00 × 10^5^	0.30
PDL, tooth follicles	50.00	0.49

**Figure 1:**
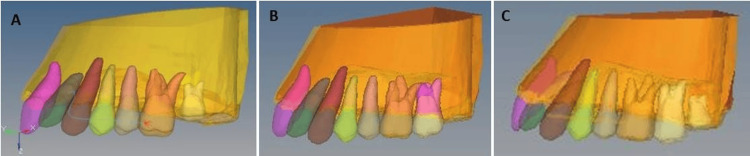
Finite element models for second and third molar eruption stages. A: The follicles of the second molars are positioned directly in front of the cervical one-third of the first molar root; B: The second molars erupted completely; C: The third molar follicles were placed at the second molar root's cervical one-third.

Previous investigations [17,26] determined the stages of the maxillary molar eruption, which were characterized as follows: Stage 1: The follicles of the second molars are positioned directly in front of the cervical one-third of the first molar root. Stage 2: The second molars erupted completely. Stage 3: The third molar follicles were placed at the second molar root's cervical one-third (Figure 1).

Appliance design

Modified Palatal Anchorage Plate

An MPAP (Jeil Medical, Seoul, Korea) was fixed in the paramedian palatal region at the sagittal level of the center of the first molar by three miniscrews (length, 8 mm; diameter, 2 mm). An SS palatal bar 1.0 mm in diameter was connected to the bands of the maxillary first molars, extending interiorly along the lingual gingival margin of the maxillary teeth. The two extended lever arms on the MPAP curve distally and each lever arm had four indentations for attachment of elastics or coils [17]. Distalization force was applied by connecting the most apical notch of the palatal plate to the hooks of the palatal bar, which is located near the center of the lingual gingival margin of the canines.

Beneslider

Two paramedian or median mini-implants (2 x 9 mm) were used to secure Beneslider in the anterior palate (Benefit system; PSM Medical Solutions, Germany/PSM North America, Indio, CA). The distalization appliance was fitted to the palatal mini-implants. A NiTi spring was compressed between the Benetube and the mobilizer, and the mobilizer was locked to produce a distalization force.

Models of six different distalization modalities for either of the appliances were installed on finite element models (FEMs) to produce 12 different models based on the mode of distalization (first molar versus en-masse maxillary arch distalization) and the eruption stages of second and third molars.

**Figure 2:**
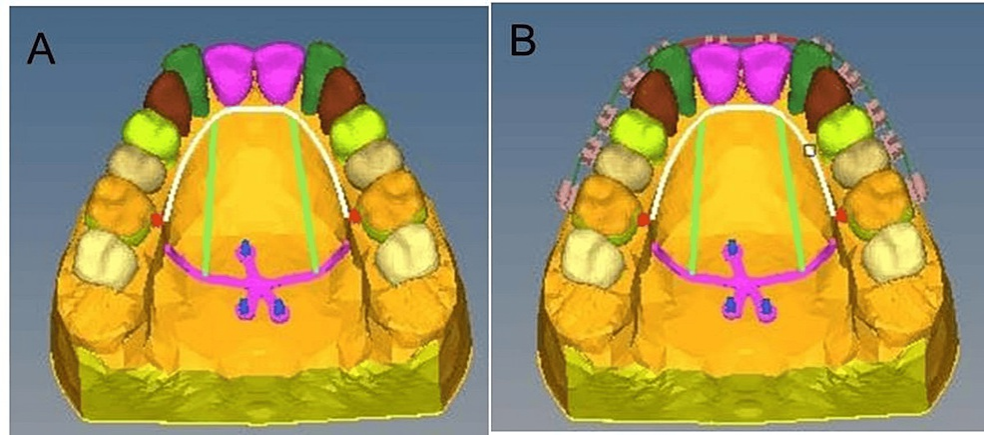
MPAP FEA model. A: MPAP FEA model without brackets for individual molar distalization; B: MPAP FEA model with brackets for EMAD. MPAP: modified palatal anchorage plate, EMAD: en-masse maxillary arch distalization, FEA: finite element analysis.

Distalization models 1, 2, and 3 with first molar distalization alone on 1, 2, and 3 eruption stages with MPAP, respectively, using 150 gm of distalizing force on each side from the most apical notch on the palatal plate to the hooks on the stainless-steel bar (Figure 2).

Distalization models 4, 5, and 6 with EMAD on 1, 2, and 3 eruption stages with MPAP and MBT 0.022-inch bracket system (3M UNITEK) attached to the teeth with a 0.019 × 0.025-inch SS archwire, cinched back distal to the first molars. 300 gm of distalizing force on each side was applied from the most apical notch on the palatal plate 10 mm apical to the archwire, vertically to the hooks on the stainless-steel bar (Figure 2).

**Figure 3:**
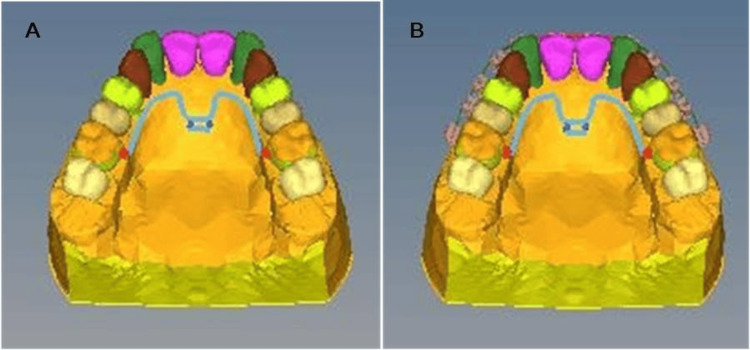
Beneslider FEA model. A: Beneslider FEA model without brackets for individual molar distalization; B: Beneslider FEA model with brackets for en-masse maxillary arch distalization. FEA: finite element analysis.

Distalization models 7, 8, and 9 with first molar distalization alone on 1, 2, and 3 eruption stages with Beneslider using 240 gm of distalizing force on each side with a nickel-titanium open-coil spring (Figure 3).

Distalization models 10, 11, and 12 with EMAD on 1, 2, and 3 stages with Beneslider and MBT 0.022-inch bracket system (3M UNITEK) attached to the teeth with a 0.019 × 0.025-inch SS archwire, cinched back distal to the first molars. A nickel-titanium open-coil spring is used to apply 500 gm of distalizing force on each side (Figure 3B). 

Boundary Conditions and 3D Coordinate System

The 3D coordinates were based on the occlusal plane: (x-axis, buccal (+) to palatal (−) direction; y-axis, anterior (+) to posterior (−) direction; and z-axis, extrusion (+) to intrusion (−) direction). The circumaxillary sutures were fixed in all directions using boundary conditions.

Non-linear static analysis was performed via ANSYS R18.1 Software. Displacements were studied for both MPAP and Beneslider on the following 12 landmarks within 12 modes of distalization in the 3D coordinate system, which includes: 1. Mesio-buccal cusp of the upper first molars, 2. Mesio-palatal cusp of the upper first molar, 3. Disto-buccal cusp of the upper first molar, 4. Disto-palatal cusp of the upper first molar, 5. Mesio-buccal cusp of the upper second molar, 6. Mesio-palatal cusp of the upper second molar, 7. Disto-buccal cusp of the upper second molar, 8. Disto-palatal cusp of the upper second molar, 9. Palatal root apex of the upper first molar, 10. Palatal root apex of the upper second molar, 11. The midpoint of the incisal edge of the central incisor, and 12. Root apex of the central incisor.

## Results

Distalization of the first molar by MPAP

Model 1

First molar displayed mesial-in rotation on the x-axis, and the palatal root apex shifted buccally. Distalization of the first molar was observed on the y-axis, with the distalization component being more in the crown than the root apex. In the z-axis, buccal cusps extruded, while both the palatal cusps and palatal root apex intruded, indicating palatal tooth rolling. Central incisor crowns moved mesially, with roots diverging distally in the x-axis; both the crown and root apex moved palatally in the y-axis, with more movement in the crown over the root apex; and incisors showed extrusion in the z-axis. 

Model 2

Movements of the first molar and central incisors were similar to model 1 in a 3D coordinate system. However, mesial-in rotation was not seen, distalization was less, and palatal rolling was more compared to model 1 on the first molar. In the x-axis, in the second molar, both the crown and the palatal root apex showed buccal displacement, with the crown showing more buccal flaring over the palatal root apex. In the y-axis, there was a distalization of the second molar, with the distalization component more in the crown compared with the root apex. In the z-axis, the second molar showed intrusion.

Model 3

**Figure 4:**
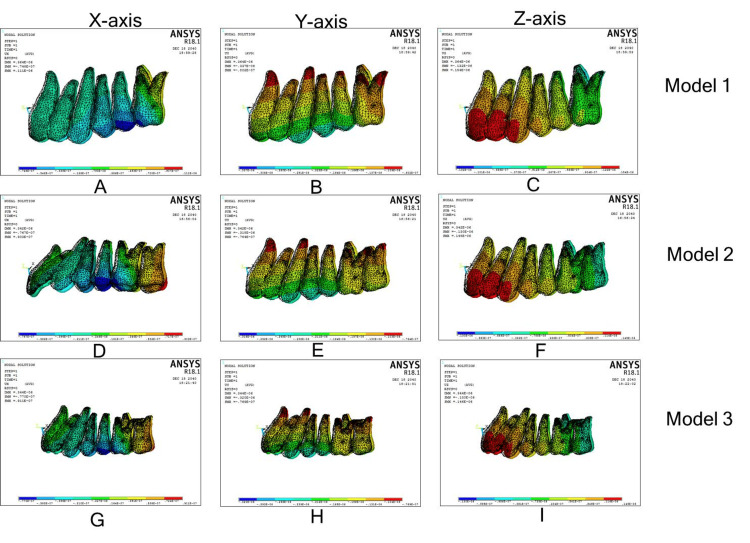
Individual molar distalization with modified palatal anchorage plate. (A)-(C): Displacement along the x, y, and z axes in model 1; (D)-(F): Displacement along the x, y, and z axes in model 2; (G)-(I): Displacement along the x, y, and z axes in model 3.

Movements of first molar, second molar, and central incisors were similar to model 2 in the 3D coordinate system (Figure 4, Table 2).

**Table 2:** 3D displacement of landmarks by distalization of the first molar with MPAP in models 1, 2, and 3. MIE: midpoint of the incisal edge, RA: root apex, MBC: mesio buccal cusp, MPC: mesio palatal cusp, DBC: distobuccal cusp, DPC: distopalatal cusp, PRA: palatal root apex, MPAP: modified palatal anchorage plate.

Model no	Δ	Central incisor		First molar					Second molar				
		MIE 375072	RA 374995	MBC 359503	MPC 356722	DBC 360045	DPC 357866	PRA 356043	MBC 36092 0	MPC 358692	DBC 36128 7	DPC 3594 02	PRA 357608
1	Δx	-6.33 × 10^-9^	3.97 × 10^-10^	-5.23 × 10^-^^8^	-5.18 × 10^-^^8^	1.31 × 10^-8^	5.26 × 10^-^​​​​​​​^8^	3.36 × 10^-^​​​​​​​^8^	-	-	-	-	-
	Δy	-2.14 × 10^-​​​​​​​7^	-8.15 × 10^-^​​​​​​​^8^	-1.93 × 10^-^​​​​​​​^7^	-2.35 × 10^-​​​​​​​7^	-1.66 × 10^-​​​​​​​7^	-2.00 × 10^-​​​​​​​7^	-1.16 × 10^-​​​​​​​7^	-	-	-	-	-
	Δz	1.27 × 10^-^​​​​​​​^7^	6.35 × 10^-^​​​​​​​^8^	1.70 × 10^-^​​​​​​​^8^	-5.56 × 10^-^​​​​​​​^8^	6.24 × 10^-^​​​​​​​^9^	-4.81 × 10^-^​​​​​​​^9^	-2.74 × 10^-^​​​​​​​^8^	-	-	-	-	-
2	Δx	-5.88 × 10^-^​​​​​​​^9^	5.13 × 10^-^​​​​​​​^10^	-5.99 × 10^-^​​​​​​​^8^	-6.48 × 10^-^​​​​​​​^8^	-4.31 × 10^-^​​​​​​​^9^	2.90 × 10^-^​​​​​​​^8^	2.93 × 10^-^​​​​​​​^8^	5.37 × 10^-^​​​​​​​^8^	6.49 × 10^-^​​​​​​​^8^	7.09 × 10^-^​​​​​​​^8^	7.72 × 10^-^​​​​​​​^8^	4.63 × 10^-^​​​​​​​^8^
	Δy	-2.03 × 10^-^​​​​​​​^7^	-7.77 × 10^-^​​​​​​​^8^	-1.92 × 10^-^​​​​​​​^7^	-2.22 × 10^-^​​​​​​​^7^	-1.71 × 10^-^​​​​​​​^7^	-1.92 × 10^-^​​​​​​​^7^	-1.15 × 10^-^​​​​​​​^7^	-1.72 × 10^-^​​​​​​​^7^	-1.98 × 10^-^​​​​​​​^7^	-1.68 × 10^-^​​​​​​​^7^	-1.88 × 10^-^​​​​​​​^7^	-1.28 × 10^-^​​​​​​​^7^
	Δz	1.19 × 10^-^​​​​​​​^7^	5.95 × 10^-^​​​​​​​^8^	2.06 × 10^-^​​​​​​​^8^	-5.27 × 10^-^​​​​​​​^8^	9.47 × 10^-^​​​​​​​^9^	-2.05 × 10^-^​​​​​​​^9^	-2.51 × 10^-^​​​​​​​^8^	-8.90 × 10^-^​​​​​​​^9^	-1.30 × 10^-^​​​​​​​^8^	-3.02 × 10^-^​​​​​​​^8^	-3.31 × 10^-^​​​​​​​^8^	-2.75 × 10^-^​​​​​​​^8^
3	Δx	-5.96 × 10^-^​​​​​​​^9^	5.09 × 10^-^​​​​​​​^10^	-5.99 × 10^-^​​​​​​​^8^	-6.45 × 10^-^​​​​​​​^8^	-3.79 × 10^-^​​​​​​​^9^	2.99 × 10^-^​​​​​​​^8^	3.00 × 10^-^​​​​​​​^8^	5.48 × 10^-^​​​​​​​^8^	6.62 × 10^-^​​​​​​​^8^	7.28 × 10^-^​​​​​​​^8^	7.92 × 10^-^​​​​​​​^8^	4.75 × 10^-^​​​​​​​^8^
	Δy	-2.04 × 10^-^​​​​​​​^7^	-7.82 × 10^-^​​​​​​​^8^	-1.94 × 10^-^​​​​​​​^7^	-2.24 × 10^-^​​​​​​​^7^	-1.72 × 10^-^​​​​​​​^7^	-1.94 × 10^-^​​​​​​​^7^	-1.16 × 10^-^​​​​​​​^7^	-1.73 × 10^-^​​​​​​​^7^	-2.00 × 10^-^​​​​​​​^7^	-1.69 × 10^-^​​​​​​​^7^	-1.89 × 10^-^​​​​​​​^7^	-1.29 × 10^-^​​​​​​​^7^
	Δz	1.20 × 10^-^​​​​​​​^7^	6.01 × 10^-^​​​​​​​^8^	2.06 × 10^-^​​​​​​​^8^	-5.30 × 10^-^​​​​​​​^8^	9.08 × 10^-^​​​​​​​^9^	-2.82 × 10^-^​​​​​​​^9^	-2.59 × 10^-^​​​​​​​^8^	-9.63 × 10^-^​​​​​​​^9^	-1.43 × 10^-^​​​​​​​^8^	-3.13 × 10^-^​​​​​​​^8^	-3.47 × 10^-^​​​​​​​^8^	-2.86 × 10^-^​​​​​​​^8^

EMAD by MPAP

Model 4

**Figure 5:**
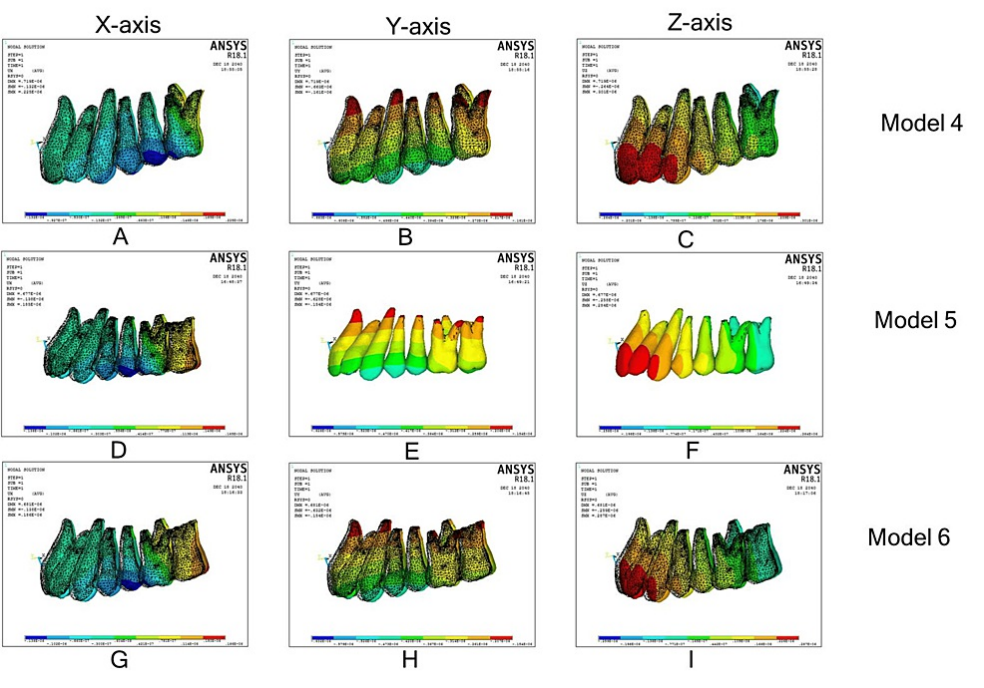
En-masse maxillary arch distalization with modified palatal anchorage plate. (A)-(C): Displacement along the x, y, and z axes in model 4; (D)-(F): displacement along the x, y, and z axes in model 5; (G)-(I): displacement along the x, y, and z axes in model 6.

Movements of first molars and central incisors were identical to model 1 in the 3D coordinate system and Models 5 and 6 first molar, second molar, and central incisors displacements in the 3D coordinate system were identical to models 2 (Figure 5; Table 3).

**Table 3:** 3D displacement of landmarks by en-masse distalization (EMAD) with MPAP in models 4, 5, and 6. MIE: midpoint of the incisal edge, RA: root apex, MBC: mesio buccal cusp, MPC: mesio palatal cusp, DBC: distobuccal cusp, DPC: distopalatal cusp, PRA: palatal root apex.

Model no	Δ	Central incisor		First molar					Second molar				
		MIE 375072	RA 374995	MBC 359503	MPC 356722	DBC 360045	DPC 357866	PRA 356043	MBC 360920	MPC 358692	DBC 361287	DPC 359402	PRA 357608
4	Δx	-1.31 × 10^−8^	8.70 × 10^−10^	-9.09 × 10^−8^	-9.43 × 10^−8^	3.13 × 10^−8^	1.06 × 10^−7^	6.71 × 10^−8^	-	-	-	-	-
	Δy	-4.22 × 10^−7^	-1.64 × 10^−7^	-3.88 × 10^−7^	-4.64 × 10^−7^	-3.39 × 10^−7^	-3.97 × 10^−7^	-2.29 × 10^−7^	-	-	-	-	-
	Δz	2.48E × 10^−7^	1.25 × 10^−7^	3.30 × 10^−8^	-1.11 × 10^−7^	1.07 × 10^−8^	-1.03 × 10^−8^	-5.41 × 10^−8^	-	-	-	-	-
5	Δx	-1.24 × 10^−8^	1.08 × 10^−9^	-1.07 × 10^−7^	-1.20 × 10^−7^	-2.48 × 10^−9^	6.12 × 10^−8^	5.89 × 10^−9^	1.08 × 10^−7^	1.30 × 10^−7^	1.41 × 10^−7^	1.53 × 10^−7^	9.15 × 10^−8^
	Δy	-4.00 × 10^−7^	-1.56 × 10^−7^	-3.85 × 10^−7^	-4.37 × 10^−7^	-3.45 × 10^−7^	-3.80 × 10^−7^	-2.28 × 10^−7^	-3.46 × 10^−7^	-3.95 × 10^−7^	-3.37 × 10^−7^	-3.75 × 10^−7^	-2.54 × 10^−7^
	Δz	2.34 × 10^−7^	1.17 × 10^−7^	4.00 × 10^−8^	-1.05 × 10^−7^	1.80 × 10^−8^	-3.95 × 10^−9^	-4.91 × 10^−8^	-1.84 × 10^−8^	-2.65 × 10^−8^	-6.10 × 10^−8^	-6.67 × 10^−8^	-5.51 × 10^−8^
6	Δx	-1.25 × 10^−8^	1.08 × 10^−9^	-1.07 × 10^−7^	-1.20 × 10^−7^	-1.45 × 10^−9^	6.29 × 10^−8^	6.04 × 10^−8^	1.10 × 10^−7^	1.32 × 10^−7^	1.44 × 10^−7^	1.57 × 10^−7^	9.38 × 10^−8^
	Δy	-4.03 × 10^−7^	-1.57 × 10^−7^	-3.88 × 10^−7^	-4.41 × 10^−7^	-3.48 × 10^−7^	-3.84 × 10^−7^	-2.30 × 10^−7^	-3.48 × 10^−7^	-3.99 × 10^−7^	-3.39 × 10^−7^	-3.78 × 10^−7^	-2.56 × 10^−7^
	Δz	2.36 × 10^−7^	1.18 × 10^−7^	4.01 × 10^−7^	-1.05 × 10^−7^	1.72 × 10^−8^	-5.49 × 10^−9^	-5.06 × 10^−8^	-1.99 × 10^−8^	-2.90 × 10^−8^	-6.32 × 10^−8^	-6.99 × 10^−8^	-5.74 × 10^−8^

Distalization of first molar by Beneslider

Model 7

On the x-axis, the first molar showed mesial-in rotation and the palatal root apex moved buccally. In the y-axis, there was a distalization of the first molar, with the distalization component similar in the crown and the root apex depicting a near translation of the first molar. In the z-axis, the first molar showed extrusion of the tooth. Central incisor crowns moved mesially, with roots diverging distally in the x-axis, both the crown and root apex moved palatally in the y-axis, with more movement in the crown over the root apex, and incisors showed extrusion in the z-axis.

Model 8

Movements of the first molar and central incisors were similar to model 7 in the 3D coordinate system, except mesial-in rotation on the first molar. With the second molar in the x-axis, both the crown and the palatal root apex showed buccal displacement, with the crown showing more buccal flaring over the palatal root apex. In the y-axis, there was a distalization of the second molar with the distalization component more in the crown compared with the root apex. In the z-axis, the second molar showed intrusion.

Model 9 

**Figure 6:**
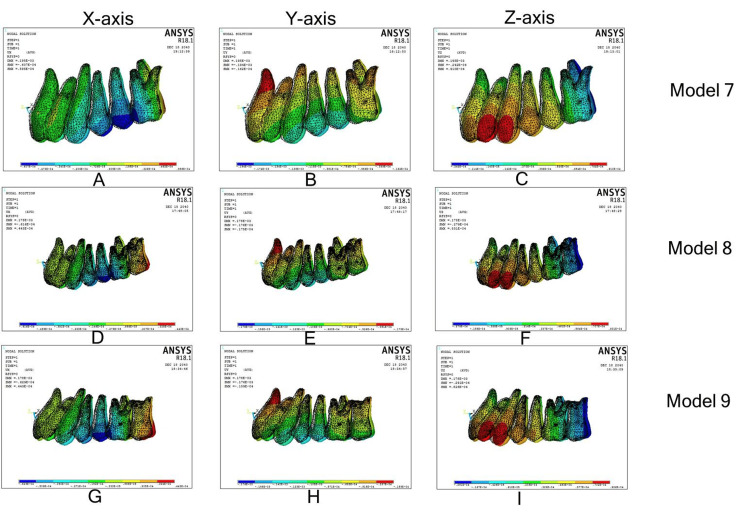
Individual molar distalization with Beneslider. (A)-(C): Displacement along the x, y, and z axes in model 7; (D)-(F): displacement along the x, y, and z axes in model 8; (G)-(I): displacement along the x, y, and z axes in model 9.

Movements of first molar, second molar, and central incisors were similar to model 8 in the 3D coordinate system (Figure 6; Table 4).

**Table 4:** 3D displacement of landmarks by distalization of the first molar with Beneslider in models 7, 8, and 9. MIE: midpoint of the incisal edge, RA: root apex, MBC: mesio buccal cusp, MPC: mesio palatal cusp, DBC: distobuccal cusp, DPC: distopalatal cusp, PRA: palatal root apex.

Model no	Δ	Central incisor		First molar					Second molar				
		MIE 375072	RA 374995	MBC 359503	MPC 356722	DBC 360045	DPC 357866	PRA 356043	MBC 360920	MPC 358692	DBC 361287	DPC 359402	PRA 357608
7	Δx	−5.26 × 10^−6^	2.60 × 10^−6^	-3.83 × 10^−5^	-1.01 × 10^−5^	9.94 × 10^−6^	4.36 × 10^−5^	3.34 × 10^−6^	-	-	-	-	-
	Δy	-8.27 × 10^−5^	-1.66 × 10^−5^	-1.24 × 10^−4^	-1.76 × 10^−4^	-1.13 × 10^−4^	-1.60 × 10^−4^	-0.13 × 10^−4^	-	-	-	-	-
	Δz	6.16 × 10^−5^	2.72 × 10^−5^	2.84 × 10^−5^	1.50 × 10^−5^	2.73 × 10^−6^	-8.96 × 10^−6^	-9.27 × 10^−6^	-	-	-	-	-
8	Δx	-4.69 × 10^−6^	2.49 × 10^−6^	-4.44 × 10^−5^	-2.37 × 10^−5^	-4.70 × 10^−6^	1.99 × 10^−5^	3.22 × 10^−6^	2.79 × 10^−5^	3.53 × 10^−5^	3.89 × 10^−5^	4.33 × 10^−5^	1.62 × 10^−5^
	Δy	-7.84 × 10^−5^	-1.79 × 10^−5^	-1.17 × 10^−4^	-1.57 × 10^−4^	-1.07 × 10^−4^	-1.41 × 10^−4^	-0.78 × 10^−4^	-1.03 × 10^−4^	-1.21 × 10^−4^	-9.84 × 10^−5^	-1.13 × 10^−4^	-6.56 × 10^−5^
	Δz	5.65 × 10^−5^	2.52 × 10^−5^	3.26 × 10^−5^	1.86 × 10^−5^	1.44 × 10^−5^	5.77 × 10^−6^	2.34 × 10^−6^	-5.39 × 10^−6^	-6.61 × 10^−6^	-2.24 × 10^−5^	-2.25 × 10^−5^	-1.48 × 10^−5^
9	Δx	-4.56 × 10^−6^	2.53 × 10^−6^	-4.57 × 10^−5^	-2.49 × 10^−5^	-5.92 × 10^−6^	1.89 × 10^−5^	3.03 × 10^−6^	2.70 × 10^−5^	3.46 × 10^−5^	3.85 × 10^−5^	4.31 × 10^−5^	1.61 × 10^−5^
	Δy	-7.59 × 10^−5^	-1.63 × 10^−5^	-1.17 × 10^−4^	-1.57 × 10^−4^	-1.07 × 10^−4^	-1.42 × 10^−4^	-0.78 × 10^−4^	-1.02 × 10^−4^	-1.24 × 10^−4^	-9.79 × 10^−5^	-1.13 × 10^−4^	-6.50 × 10^−5^
	Δz	5.54 × 10^−5^	2.45 × 10^−5^	3.26 × 10^−5^	1.81 × 10^−5^	1.42 × 10^−5^	5.06 × 10^−6^	2.98 × 10^−6^	-5.73 × 10^−6^	-7.56 × 10^−6^	-2.30 × 10^−5^	-2.37 × 10^−5^	-1.57 × 10^−5^

EMAD with Beneslider

Model 10

On the x-axis, the first molar showed mesial-in rotation and the palatal root apex moved buccally. In the y-axis, there was a distalization of the first molar with the distalization component more in the crown compared with the root apex. In the z-axis, the distopalatal cusp and palatal root apex intruded, while the rest of the tooth showed extrusion. Central incisor crowns moved mesially, with roots diverging distally in the x-axis; both the crown and root apex moved palatally in the y-axis, with more movement in the crown over the root apex; and incisors showed extrusion in the z-axis. 

Model 11

It had similar displacements on the first molar, and central incisors in a 3D coordinate system, except mesial-in rotation on the first molar. In the x-axis, in the second molar, both the crown and the palatal root apex showed buccal displacement with the crown showing a more buccal flaring over the palatal root apex. In the y-axis, there was a distalization of the second molar with the distalization component more in the crown compared with the root apex. In the z-axis, the second molar showed intrusion.

Model 12

**Figure 7:**
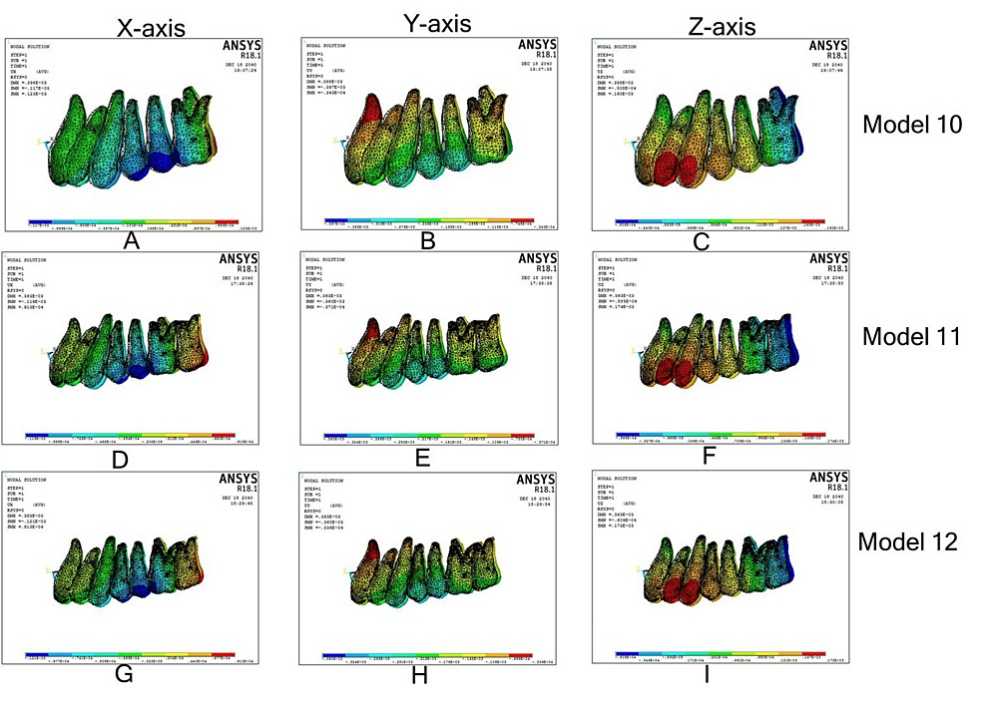
En-masse maxillary arch distalization with Beneslider. (A)-(C): Displacement along the x, y, and z axes in model 10; (D)-(F): displacement along the x, y, and z axes in model 11; (G)-(I): displacement along the x, y, and z axes in model 12.

It had identical displacements to model 11 in the 3D coordinate system (Figure 7; Table 5).

​​​​​​​

**Table 5:** 3D displacement of landmarks by en-masse distalization with Beneslider in models 10, 11, and 12. MIE: midpoint of the incisal edge, RA: root apex, MBC: mesio buccal cusp, MPC: mesio palatal cusp, DBC: distobuccal cusp, DPC: distopalatal cusp, PRA: palatal root apex.

Model no	Δ	Central incisor		First molar					Second molar				
		MIE 375072	RA 374995	MBC 359503	MPC 356722	DBC 360045	DPC 357866	PRA 356043	MBC 360920	MPC 358692	DBC 361287	DPC 359402	PRA 357608
10	Δx	-1.60 × 10^−5^	5.57 × 10^−6^	-7.02 × 10^−5^	-1.42 × 10^−5^	2.49 × 10^−5^	9.22 × 10^−5^	7.20 × 10^−6^	-	-	-	-	-
	Δy	-1.75 × 10^−4^	-3.51 × 10^−5^	-2.59 × 10^−4^	-3.62 × 10^−4^	-2.39 × 10^−4^	-3.31 × 10^−4^	-1.08 × 10^−4^	-	-	-	-	-
	Δz	1.28 × 10^−4^	5.53 × 10^−5^	5.83 × 10^−5^	3.16 × 10^−5^	4.52 × 10^−6^	-1.89 × 10^−5^	-1.87 × 10^−5^	-	-	-	-	-
11	Δx	-1.45 × 10^−5^	5.31 × 10^−6^	-8.34 × 10^−5^	-4.25 × 10^−5^	-5.08 × 10^−6^	4.42 × 10^−5^	7.25 × 10^−6^	5.93 × 10^−5^	-7.41 × 10^−5^	8.07 × 10^−5^	8.96 × 10^−5^	3.32 × 10^−5^
	Δy	-1.66 × 10^−4^	-3.78 × 10^−5^	-2.43 × 10^−4^	-3.23 × 10^−4^	-2.24 × 10^−4^	-2.92 × 10^−4^	-1.14 × 10^−4^	-2.14 × 10^−4^	-2.51 × 10^−4^	-2.06 × 10^−4^	-2.35 × 10^−4^	-1.35 × 10^−4^
	Δz	1.17 × 10^−4^	5.11 × 10^−5^	6.71 × 10^−5^	3.92 × 10^−5^	2.92 × 10^−5^	1.22 × 10^−5^	-3.98 × 10^−6^	-1.17 × 10^−5^	-1.4 × 10^−5^	-4.71 × 10^−5^	-4.71 × 10^−5^	-3.08 × 10^−5^
12	Δx	-1.45 × 10^−5^	5.41 × 10^−6^	-8.62 × 10^−5^	-4.51 × 10^−5^	-7.56 × 10^−6^	4.21 × 10^−5^	6.85 × 10^−6^	5.74 × 10^−5^	7.27 × 10^−5^	8.00 × 10^−5^	8.92 × 10^−5^	3.30 × 10^−5^
	Δy	-1.61 × 10^−4^	-3.45 × 10^−5^	-2.43 × 10^−4^	-3.23 × 10^−4^	-2.24 × 10^−4^	-2.92 × 10^−4^	-1.13 × 10^−4^	-2.14 × 10^−4^	-2.51 × 10^−4^	-2.04 × 10^−4^	-2.35 × 10^−4^	-1.34 × 10^−4^
	Δz	1.15 × 10^−4^	4.97 × 10^−5^	6.70 × 10^−5^	3.81 × 10^−5^	2.88 × 10^−5^	1.07 × 10^−5^	-5.28 × 10^−6^	-1.24 × 10^−5^	-1.6 × 10^−5^	-4.84 × 10^−5^	-4.95 × 10^−5^	-3.27 × 10^−5^

## Discussion

Treatment regimens have taken a paradigm shift in class II malocclusions towards non-extraction with the advent of temporary anchorage device (TAD)-assisted molar distalization. Evidence from the literature confirms that the degree of distal tip of first molars with palatally anchored molar distalizers was significantly lesser compared to conventional appliances and buccal mini-implant-based molar distalization appliances. However, the timing of molar distalization remains controversial, specifically whether the distalization should be done before or after the eruption of the second molar. The amount of distal movement of the maxillary first molars was substantially greater and anchorage loss was significantly lower when second molars had not erupted. Molar distalization time was greatly reduced, and the movement rate was increased twofold. A larger root surface area is an element behind the resistance to distalization in the presence of a second molar [19]. 

During molar distalization, a tooth bud functions on the neighboring mesial tooth similar to a fulcrum. For example, if the maxillary molar's rotation axis is near the trifurcation of its root, a second molar bud likely causes noticeable distal tipping of the first molar if it is apical to the level of the rotation axis. To reduce the tipping of the first molar, one should either apply extra torque to the first molar or wait for the second molar eruption. Prior germectomy of the third molar is necessary if distalization of the first and second molars is performed concurrently [17]. A systematic review suggested that second and third molars have little impact on first molar distalization. However, the above evidence was established from studies comparing conventional molar distalizers (buccal and palatal), mini-implant-based buccal distalizers, and palatal appliances such as pendulum appliances and MPAP [22].

Our study is the first of its kind comparing the efficacy of two palatal implant-based molar distalization appliances (MPAP vs Beneslider) and the influence of second and third molars on first molar distalization with Finite Element Analysis during individual molar distalization as well as EMAD.

Displacement pattern with individual first molar distalization

During the first molar distalization with the second molar follicle at the cervical one-third of the first molar root, MPAP showed molar mesial in rotation, palatal rolling, and distalization with a significant distal tip. With Beneslider, the first molar showed identical movements to MPAP in the transverse plane; however, it showed near bodily distalization in the sagittal plane and extrusion in the vertical plane. With a fully erupted second molar as well as a third molar follicle in the alveolar crest MPAP showed a first molar palatal rolling and distalization with a distal tip but the tip was lesser in magnitude compared to the second molar follicular stage. The second molar showed buccal flaring, a distal tip, and intrusion, while Beneslider showed a distal translation of the first molar along with extrusion and palatal rolling. The second molar with Beneslider showed similar movements as with MPAP in stages 2 and 3 of the molar eruption sequence. Central incisors extruded and displaced palatally in all stages of the molar eruption sequence with either of the appliances. Results of the current study were corroborated by past studies by Kook et al. [12], who reported a distal tip of 3.4 degrees, and Park et al. [27], who reported 4.36 degrees of the distal tip in the first molar during distalization with MPAP. Clinical trials by Kook et al. [12] reported similar incisal retraction and extrusion as observed in our FEM study. However, Kang et al. [23] and Yu et al. [13] reported contrasting results with MPAP in a model with a second molar in the follicle stage. Kang et al. [23] showed distalization with more movement in the roots than at the first molar crowns, intrusion of the molar, central incisor, and flaring of central incisors. The plausible explanation behind this finding could be the difference in the finite element models. Kang et al. [23] and Yu et al. [13] used a FEM model with a 0.022-inch bracket system attached to the teeth and a 0.019 x 0.025” stainless steel archwire tied through frictionless translation, while the model used during individual molar distalization in this study was devoid of orthodontic appliances with either of the appliances FEM model with individual molar distalization in the current study was created to emulate the clinical scenario of Class II malocclusion with crowded maxillary anterior teeth, requiring molar distalization for increasing the arch circumference. Results with Beneslider were in line with conclusions of a clinical study by Nienkemper et al. [28], who reported a bodily displacement of centroid of the first molar by 3.60 mm and extrusion by 2 mm after the first molar displacement with Beneslider in individuals with second molars in the bud stage. Distal vector of force from the interconnected transseptal fibers in maxillary dentition would have caused the palatal extrusive displacement of incisors, even though the distalization force was applied on individual molars. Our results from the follicular second molar model strongly justify Kinzinger et al. [17] theory; a distal tooth bud acts as a fulcrum for distalizing a molar, and because the axis of rotation of the maxillary molar is near the trifurcation of its roots, a second molar tooth bud will cause pronounced distal tipping of the first molar if the second molar has not erupted beyond the vertical level of the axis of rotation. The reduced tip in the presence of a fully erupted second molar supports the notion that the fulcrum of rotation of the first molar is reduced with the complete eruption of the second molar. Our observations on the second molar movement are in line with previous studies by Yu et al. [13] and Kang et al. [23], who reported a lateral widening and intrusion of the second molars during first molar distalization with MPAP. Results of this study showed a pronounced distal displacement and distal translation of the first molar during different eruption stages of maxillary second and third molars; however, a reduced rate of displacement and a distal tip of the first molar with MPAP. A plausible explanation could be in our study, we used Beneslider with BENEtube, standard (33-54535) of 4.5 mm height and this orients the distalizing force close to the center of resistance of the first molar giving the molar a translatory distal movement. However, with MPAP an angulated distal force vector from the hooks on the palatal bar connecting bilateral molars to the most apical notch on the palatal plate could have resulted in a distal tip on the first molar. The magnitude of distalization force; 150 g with MPAP and 240 g with Beneslider (individual molar distalization), 300 g with MPAP, and 500 g with Beneslider (en-masse maxillary arch distalization) applied according to the manufacturer's recommendations could also be a reason behind the differential rate of distalization between the appliances.

Displacement pattern with EMAD

The current study showed a distal tip of the first molars, with both appliances; however, this was less pronounced with the eruption of second molars. Mesial in rotation, palatal rolling of first molars, buccal flaring and intrusion of second molars with both appliances were similar to individual molar distalization. During EMAD, a distal tip on the molar is more pronounced and seen with either of the appliances and during all stages of eruption and this could be due to the cervical location of the force vector relative to the CR of the maxillary dental arch. 

Limitations

The actual biological response might vary from the results of the FEM model, and hence the need for a clinical trial to establish clinical evidence on this orthodontic treatment regimen is necessary as part of future studies.

## Conclusions

Outcomes from the current FEM study conclude: Beneslider showed distal translation and increased rate of displacement over MPAP with individual molar distalization, during all stages of the maxillary molar eruption sequence. Distalization of the first molar was unaffected by the presence of a fully erupted second molar and third molar follicle; however, the degree of the distal tip on the first molar was reduced with the complete eruption of the second molar. Palatal rolling and extrusion of maxillary first molars was seen with both MPAP and Beneslider. Fully erupted second molars showed buccal flaring and intrusion and central incisors showed palatal displacement and extrusion during individual and en-masse maxillary arch distalization with both MPAP and Beneslider.
